# Risk Factors for Stunting in Children under the Age of 5 in Rural Guatemalan Highlands

**DOI:** 10.5334/aogh.2433

**Published:** 2020-02-03

**Authors:** Emily A. Kragel, Alexandra Merz, Dylan M. N. Flood, Kelley E. Haven

**Affiliations:** 1 East Carolina University, US; 2 Duke University, US

## Abstract

**Background:**

Previously, a study conducted by UNICEF found that malnutrition affects approximately 80% of the indigenous children in Guatemala.

**Objective:**

Identify prevalence and risk factors for stunted growth in communities surrounding Lake Atitlán, Guatemala.

**Methods:**

Height-for-age measurements of children under the age of five, N = 84, determined stunting prevalence and presumed burden of malnutrition in this region of the Guatemalan highlands. Mothers of a subset of this sample, N = 29, were interviewed to assess factors contributing to stunting. Analysis assessed the following risk factors: inadequate nutrition, increased infectious disease risk, high rate self-report illness, inadequate breastfeeding, and inadequate utilization of prenatal care.

**Findings:**

The majority of children under the age of five were stunted (65.6%) and likely malnourished. ANOVA analysis showed significant differences in mean height-for-age Z scores (HAZs) between groups with and without adequate nutrition (F = 7.069, p = 0.013), as well has with and without high rates of self-report illness (F = 6.894, p = 0.014). Both groups with inadequate nutrition (mean HAZs = –2.9, 95% CI = [–3.58, –2.24]) and high rates of self-report illness (mean HAZs = –2.8, 95% CI = [–3.13, –2.38]) had mean HAZs that are indicative of stunting. No other risk factors were associated with stunting.

**Conclusion:**

These pilot study results offer methods by which to obtain baseline data for assessing nutritional and public health interventions to improve stunting and malnutrition status as well as the health outcomes of children in rural, indigenous communities.

## Introduction

Stunting, low height-for-age, is an indicator of malnutrition [1]. The following factors are known to contribute to stunting: inadequate nutrition including lack of dietary diversity, poor maternal health and nutrition, high rates of infectious disease, and inadequate breastfeeding practices [2,3,4,5,6,7,8,9,10,11,12,13]. The WHO Conceptual Framework on Childhood Stunting identifies the household and family, breastfeeding techniques, infection, and complementary feeding practices, as working in conjunction as causative factors for stunting [14]. Complementary feedings is defined as the set of feeding methods implemented once breast milk alone becomes insufficient (6–24 months) [15]. The three factors identified within complementary feeding as causes of stunting are: poor quality foods, inadequate feeding practices, and lack of food and water safety (leading to infection) [14]. Studies have also found elevated stunting rates in households with three or more children under the age of five, 5–7 household members, and mothers who attended less than four antenatal care services during pregnancy [16]. Recent research has emphasized the impact of residential area on stunting. An association between residential area and undernutrition was found in Vietnam and regions of Ethiopia [17,18]. Similarly, in Indonesia, stunting was found to significantly differ across region, with higher rates in Eastern than Western parts of Indonesia [19]. This is likely due to differences in urbanization and location of the majority of rural indigenous Indonesian populations.

A child is most susceptible to stunting in the first 1,000 days of life, and adequate nutrition and control of infection within this critical period is crucial [20]. Cessation of stunting risk factors within the first 6–24 months of a child’s life could have significant effects on development at the individual and community levels [20]. Consequences of malnutrition include impaired physical and cognitive development, susceptibility to infectious disease, and risk of obesity and chronic disease in adulthood [9,10,11,21,22,23,24]. In addition to its debilitating effects, stunting is detrimental to economic productivity [25]. For every 1% loss in adult height due to stunting, another 1.4% of economic productivity is lost. Similarly, countries’ GDPs have displayed a 3% reduction due to stunting [25]. The World Health Organization classifies regions with at least 40% of the population stunted as serious stunting. Sustainable Development Goal 2 target 2 claims to, “*By 2030, end all forms of malnutrition, including achieving, by 2025, the internationally agreed targets on stunting and wasting in children under five years of age, and address the nutritional needs of adolescent girls, pregnant and lactating women and older persons*” [26]. However, at this rate by the year 2025, approximately 127 million children under the age of five will be stunted [27]. The World Health Organization’s goal is to reduce this number to 100 million. This 40% reduction goal cannot be accomplished with the lack of initiatives present today, especially with regard to indigenous populations [27].

Approximately 40% of the Guatemalan population identifies as indigenous [28]. Almost 50% of Guatemalan school-age children suffer from chronic malnutrition [29,30], and 80% of indigenous children are chronically malnourished [31]. Assessment of the prevalence of stunting and risk factors for stunting could help inform public health initiatives in the rural Guatemalan highlands and be applied to other indigenous rural areas where stunting has become increasingly more prevalent. Malnutrition is an important issue for ensuring health equity and improving health outcomes in the indigenous population. The data collected in this study serves as baseline data and will determine which risk factors are actually contributing to stunting in indigenous populations to inform public health initiatives and health interventions to combat a problem that has been challenging indigenous rural populations in Guatemala for decades.

## Methods

The study design was approved by East Carolina University IRB- UMCIRB 18-000418. Informed consent was obtained verbally. Community members informed survey design to ensure appropriate translation and cultural competency. A translator was utilized; all participants were indigenous and spoke Kaqchikel. Participants were recruited via community announcements from four remote villages surrounding Lake Atitlán. N = 84 children under the age of five were recruited. Height and age measurements of children under the age of five were taken. N = 29 women over the age of 18 with children under the age of five were selected for interviews assessing risk factors for stunting (Table 1). Women were selected for interviews based on their desire or ability to answer questions pertaining to their prenatal care, their breastfeeding practices, their child’s health and their child’s nutritional intake. The framework method was used to determine coding criteria for the subjective measures of adequate prenatal care and increased infectious disease risk [32]. Height-for-age Z scores (HAZs) were determined using WHO Child Growth Standards [1]. Survey responses were coded into categories (Table 1). Children with HAZs <2 St. Dev. and <3 St. Dev. below WHO Child Growth Standards Median were classified as stunted and severely stunted, respectively [1]. ANOVA analysis determined association between risk factors and HAZs.

**Table 1:** Description of risk factors and categorization of responses.

Risk factor	Measures	Criteria	Example descriptive results

Inadequate prenatal care	Utilization of healthcare services during pregnancy and prenatal dietary supplementation	Did not seek any prenatal care from clinic or hospital, inadequate nutrition during pregnancy	Only took additional folate and vitamin supplements for 1 month Did not seek prenatal care from clinic or hospital
Inadequate nutrition	24-hour food recall for child’s diet using USDA automated multiple-pass method [33]	Infant and Young Child Minimum Acceptable Diet indicator for children under the age of 2 (WHO 2009) OR dietary diversity score ≤4 for children between the ages of 2–5 [34]	Did not eat any sources of protein Did not eat any dairy products
Increased infectious disease risk	Utilization of healthcare services, perceived barriers to care, and infectious disease risk factor assessment (clean water practices, soap availability, animal contact, disease vectors)	1. Poor access to care: no utilization of healthcare facility, >2 hours travel distance to clinic or hospital, inability to afford medical visit or medication, or description of lack of trust or poor care and 2. Increased risk of disease: inappropriate soap use, no access to potable water, more than weekly contact with animals, or no toilet in house	Use of a latrine Animals living in household Not changing filter on water source Description of not being able to get medications from public health post
High rate self-report illness	Self-report of frequent illness	Report by mother of child being sick >1 time a month	Fever Vomiting Stomach pain
Inadequate breast-feeding	Onset and duration of breastfeeding and timing of complementary feeding initiation	Inappropriate onset or duration of breastfeeding (<birth-2 years) or inadequate timing of transition to other foods (>6 months) [35]	Exclusive breastfeeding for longer than 6 months

## Results

A total of 84 children completed height and age measurements, a total of 31 women completed interviews. Reasons for poor attrition include unwillingness to answer sensitive questions or absence of mother due to work. Of the 84 children, 27 had siblings included in the dataset representing a total of 12 households. The average age of children measured was 2.6 years and 58.3% of children were female; 65.6% of children were stunted and 24.1% were severely stunted. The mean HAZs was –1.83, standard deviation = 1.48. Male and female children showed similar HAZs with mean values of –1.88 and –1.8, respectively. Within the 12 households that had multiple children participate, 58.3% of children were found to all be stunted or not stunted. A low rate of prenatal care utilization (21.9%) was found. 93.5% breastfed, but 37.5% of breastfeeding practices did not meet the WHO standards [21]. A high rate of increased infectious disease risk (78.1%) was also found.

Participants with inadequate nutritional intake had a significantly lower mean HAZs (Table 2). The mean HAZs for participants with inadequate nutrition (–2.9) was almost in the severely stunted category (less than –3), while participants with adequate nutrition had a mean HAZs indicative of no stunting (greater than –2). This suggests that children with inadequate nutrition were more likely to be stunted. With regard to nutritional intake, participants lacked dietary diversity but had adequate feeding frequency. There were low consumption levels of organ meat (0%), dark leafy greens (0%), fish and seafood (7%), milk and milk products (24%), eggs (31%), and meat and poultry (44%). Additionally, participants with high rates of self-report illness had a significantly lower mean HAZs (Table 2). Once again, the mean HAZs for participants with high rate self-report illness (–2.8) was almost in the severely stunted category, while the mean HAZs for low self-report illness was in the not stunted range. The other identified risk factors showed no significant differences in HAZs (Table 2).

**Table 2:** Comparison of ANOVA results summarizing differences in mean HAZs based on risk factors.

Group	Mean HAZs	Std. Dev.	95% CI	F	P

Adequate prenatal care	–2.1	1.60	(–3.58, –0.62)	0.010	0.921
Inadequate prenatal care	–2.2	1.28	(–2.73, –1.59)		
Adequate nutrition	–1.7	1.32	(–2.33, –1.02)	7.069	0.013
Inadequate nutrition	–2.9	1.00	(–3.58, –2.24)		
No increased infectious disease risk	–2.1	0.26	(–2.65, –1.57)	0.61	0.807
Increased infectious disease risk	–2.3	0.71	(–4.10, –0.43)		
Low rate self-report illness	–1.6	1.56	(–2.44, –0.71)	6.894	0.014
High rate self-report illness	–2.8	0.65	(–3.13, –2.38)		
Adequate breastfeeding	–2.24	1.33	(–2.91, –1.58)	0.257	0.616
Inadequate breastfeeding	–1.98	1.38	(–2.91, –1.05)		

## Conclusion

The high prevalence of stunting in this region warrants intervention. The results indicate that nutrition could be the most important factor. However, interventions to combat stunting must be multidisciplinary and context-specific, as no singular solution exists. It is necessary to note that this pilot study was carried out on a small scale, and more research must be conducted with a larger sample size to confirm the validity and accuracy of the results. Additionally, a randomized method for participant selection should be implemented to reduce selection bias and ensure accuracy of results and generalizability of findings. Nevertheless, the findings that inadequate nutrition, specifically a lack of dietary diversity, combined with high self-reports of illness leads to increased stunting in this indigenous population warrants concern and ultimately further research. This issue is of particular important as it is an issue that has prevailed in this region for decades without significant improvement.

The association between high rates of self-report illness and low mean HAZs could be misleading. This could indicate that either high rates of illness are contributing to stunting, or that compromised immune status as a result of malnutrition is contributing to high rates of illness. Malnutrition is associated with compromised immune status, and there exists a synergistic relationship between infectious disease and malnutrition [9,10,11,36,37]. However, since increased infectious disease risk was not significantly associated with lower HAZs, it is likely that stunting and potential malnutrition are leading to increased self-report illness. However, infectious disease risk factors are still important to consider in the future with larger investigations and planning of public health interventions.

Previously, it was estimated that 2.5 million Guatemalans were at risk of food insecurity, with a lack of dietary diversity and inadequate meal frequency documented in the rural indigenous population [38,39]. From the results of this study, it seems that food insecurity and lack of dietary diversity are still present in this region and likely have a negative impact on rates of stunting. While there are methodological limitations in studying nutritional intake, namely the reliability and willingness of participant response, this limitation was mitigated by assessing dietary diversity and food frequency rather than caloric intake. It is possible that participant responses to survey questions had social desirability bias. Nevertheless, this pilot study showed that these methods are feasible and can be applied to serve as an instrument for obtaining baseline data to assess efficacy of public health interventions.

Potential community interventions to increase dietary diversity include empowerment of local women and families with resources to increase access to low cost nutritional sources. Alternatively, community interventions could improve sanitation or factors contributing to rates of infectious disease. Future interventions should be guided by community participatory development to ensure feasibility and relevance. Future research conducted with a larger and randomized sample should be utilized in this manner to collect and analyze baseline data as well as interval data to ensure that interventions are effective. Given the nature of global health work and the lack of resources often available in rural, indigenous communities it is imperative that these resources are utilized effectively and are demonstrating a positive impact through research.
